# 2,2′-{1,1′-[Pentane-1,5-diylbis(oxy­nitrilo)]diethylidyne}di-1-naphthol

**DOI:** 10.1107/S1600536809016584

**Published:** 2009-05-14

**Authors:** Yin-Xia Sun, Jian-Chao Wu, Wen-Kui Dong, Shang-sheng Gong, Jun-Feng Tong

**Affiliations:** a School of Chemical and Biological Engineering, Lanzhou Jiaotong University, Lanzhou 730070, People’s Republic of China

## Abstract

The title compound, C_29_H_30_N_2_O_4_, adopts a distorted *Z* configuration with respect to the oxime group, which is almost coplanar with the adjacent naphthalene ring [dihedral angle = 4.11 (2)°]. There is one half-mol­ecule in the asymmetric unit, with a crystallographic twofold rotation axis passing through the central C atom of the –CH=N—O-(CH)_5_—O—N=CH– bridge. Within the mol­ecule, the dihedral angle formed by the two naphthalene rings is 79.08 (3)°, and there are two intra­molecular O—H⋯N hydrogen bonds.

## Related literature

The condensation of primary amines with active carbonyl compounds can yield Schiff bases, see: Atwood & Harvey (2001 ▶); Casellato & Vigato (1977 ▶). For related structures, see: Dong *et al.* (2008*a* ▶,*b* ▶,*c* ▶, 2009 ▶); Shi *et al.* (2007 ▶); Yeap *et al.* (2008 ▶).
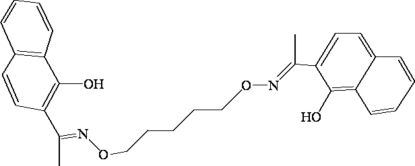


## Experimental

### 

#### Crystal data

C_29_H_30_N_2_O_4_
*M* *_r_* = 470.55
Monoclinic, 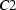

*a* = 14.0683 (12) Å
*b* = 4.5659 (7) Å
*c* = 19.048 (2) Å
β = 97.321 (1)°
*V* = 1213.6 (2) Å^3^
*Z* = 2
Mo *K*α radiation
μ = 0.09 mm^−1^
*T* = 298 K
0.49 × 0.45 × 0.21 mm

#### Data collection

Bruker SMART CCD area-detector diffractometer
Absorption correction: multi-scan (*SADABS*; Sheldrick, 1996 ▶) *T*_min_ = 0.959, *T*_max_ = 0.982
3170 measured reflections
1218 independent reflections
814 reflections with *I* > 2σ(*I*)
*R*_int_ = 0.034

#### Refinement

*R*[*F* ^2^ > 2σ(*F* ^2^)] = 0.043
*wR*(*F* ^2^) = 0.111
*S* = 1.02
1218 reflections
159 parameters
1 restraint
H-atom parameters constrained
Δρ_max_ = 0.11 e Å^−3^
Δρ_min_ = −0.13 e Å^−3^

### 

Data collection: *SMART* (Siemens, 1996 ▶); cell refinement: *SAINT* (Siemens, 1996 ▶); data reduction: *SAINT*; program(s) used to solve structure: *SHELXS97* (Sheldrick, 2008 ▶); program(s) used to refine structure: *SHELXL97* (Sheldrick, 2008 ▶); molecular graphics: *SHELXTL* (Sheldrick, 2008 ▶); software used to prepare material for publication: *SHELXTL*.

## Supplementary Material

Crystal structure: contains datablocks global, I. DOI: 10.1107/S1600536809016584/at2776sup1.cif

Structure factors: contains datablocks I. DOI: 10.1107/S1600536809016584/at2776Isup2.hkl

Additional supplementary materials:  crystallographic information; 3D view; checkCIF report

## Figures and Tables

**Table 1:** Hydrogen-bond geometry (Å, °)

*D*—H⋯*A*	*D*—H	H⋯*A*	*D*⋯*A*	*D*—H⋯*A*
O2—H2⋯N1	0.82	1.83	2.552 (3)	145

**Fig. 1.**
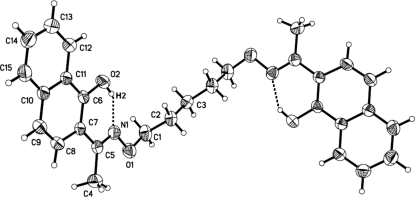
The molecular structure of the title compound with atom-numbering scheme [symmetry code: (#1) - x, y, -z]. Displacement ellipsoids for non-hydrogen atoms are drawn at the 30% probability level.

## supplementary crystallographic information

### Comment

The condensation of primary amines with active carbonyl compounds can yield Schiff bases (Casellato & Vigato, 1977; Atwood & Harvey, 2001) that are still now regarded as one of the most potential group of chelators for facile preparations of metallo-organic hybrid materials (Yeap *et al.*, 2008). Many Schiff base complexes have been structurally characterized, but at present, a new class of salen-type bisoxime compounds have been synthesized by using an *O*-alkyloxime unit (–CH═N—O-(CH)_n_—O—N═CH–) instead of the (–CH═N-(CH)_n_—N═CH–) group (Dong *et al.*, 2008*a*,*b*), the large electronegativity of O atoms is expected to affect strongly the electronic properties of N_2_O_2_ coordination sphere. Herein, we report on the synthesis and crystal structure of 2,2'-{1,1'-[pentane-1,5-diylbis(oxynitrilo)]diethylidyne}dinaphthol, (I), (Fig. 1).

The single-crystal structure of the title compound (I) is built up by only the C_29_H_30_N_2_O_4_ molecule, in which all bond lengths are in normal ranges. It adopts a distorted *Z* configuration with respect to the bisoxime group which is coplanar with the adjacent naphthalene ring (Dong *et al.*, 2008*c*). The structure of (I) reveals the C1, C2, C3, C2# and C1# atoms in the (–CH═N—O-(CH)_5_—O—N═CH–) bridge lying on a plane. The dihedral angle formed by the two naphthalene rings in each molecule of the title compound is about 79.08°. Within each half of the molecule, the dihedral angle formed by the plane of the (–(CH)_5_–) bridge and the adjacent naphthalene ring is about 62.74°. There are two intramolecular hydrogen bonds, O2—H2···N1 (Table 1), indicating a strong hydrogen-bonding interaction.

### Experimental

2,2'-{1,1'-[Pentane-1,5-diylbis(oxynitrilo)]diethylidyne}dinaphthol was synthesized according to an analogous method reported earlier (Shi *et al.*, 2007; Dong *et al.*, 2009). To an ethanol solution (5 ml) of 2-acetyl-1-naphthol (374.7 mg, 2.02 mmol) was added dropwise an ethanol solution (3 ml) of 1,5-bis(aminooxy)pentane (133.5 mg, 0.94 mmol). The mixture solution was stirred at 328–333 K for 70 h. After cooling to room temperature, the precipitate was filtered off, and washed successively three times with ethanol. The product was dried *in vacuo* and purified by recrystallization from ethanol to yield 367.5 mg (yield, 83.1%) of solid; m.p. 394–396 K. Colourless needle-like single crystals suitable for X-ray diffraction studies were obtained by slow evaporation from a solution of chloroform/methanol (1:1) of 2,2'-{1,1'-[pentane-1,5-diylbis(oxynitrilo)]diethylidyne}dinaphthol at room temperature for about three weeks. Analysis calculated for C_29_H_30_N_2_O_4_: C 74.02, H 6.43, N 5.99 (%); Found: C 74.05, H 6.39, N 6.07(%).

### Refinement

H atoms were treated as riding atoms with distances C—H = 0.96 (CH_3_), C—H = 0.97 (CH_2_), 0.93 Å (CH), O—H = 0.82 Å [*U*_iso_(H) = 1.2 *U*_eq_(C) and 1.5 *U*_eq_(C,O)].

### Crystal data

**Table:**

C_29_H_30_N_2_O_4_	*F*(000) = 500
*M**_r_* = 470.55	*D*_x_ = 1.288 Mg m^−^^3^
Monoclinic, *C*2	Mo *K*α radiation, λ = 0.71073 Å
Hall symbol: C 2y	Cell parameters from 907 reflections
*a* = 14.0683 (12) Å	θ = 2.9–22.5°
*b* = 4.5659 (7) Å	µ = 0.09 mm^−^^1^
*c* = 19.048 (2) Å	*T* = 298 K
β = 97.321 (1)°	Block-like, yellow
*V* = 1213.6 (2) Å^3^	0.49 × 0.45 × 0.21 mm
*Z* = 2	

### Data collection

**Table:**

Bruker SMART CCD area-detector diffractometer	1218 independent reflections
Radiation source: fine-focus sealed tube	814 reflections with *I* > 2σ(*I*)
graphite	*R*_int_ = 0.034
φ and ω scans	θ_max_ = 25.0°, θ_min_ = 2.2°
Absorption correction: multi-scan (*SADABS*; Sheldrick, 1996)	*h* = −16→9
*T*_min_ = 0.959, *T*_max_ = 0.982	*k* = −5→5
3170 measured reflections	*l* = −21→22

### Refinement

**Table:**

Refinement on *F*^2^	Primary atom site location: structure-invariant direct methods
Least-squares matrix: full	Secondary atom site location: difference Fourier map
*R*[*F*^2^ > 2σ(*F*^2^)] = 0.043	Hydrogen site location: inferred from neighbouring sites
*wR*(*F*^2^) = 0.111	H-atom parameters constrained
*S* = 1.02	*w* = 1/[σ^2^(*F*_o_^2^) + (0.057*P*)^2^] where *P* = (*F*_o_^2^ + 2*F*_c_^2^)/3
1218 reflections	(Δ/σ)_max_ < 0.001
159 parameters	Δρ_max_ = 0.11 e Å^−^^3^
1 restraint	Δρ_min_ = −0.13 e Å^−^^3^

### Special details

**Table:**

Geometry. All e.s.d.'s (except the e.s.d. in the dihedral angle between two l.s. planes) are estimated using the full covariance matrix. The cell e.s.d.'s are taken into account individually in the estimation of e.s.d.'s in distances, angles and torsion angles; correlations between e.s.d.'s in cell parameters are only used when they are defined by crystal symmetry. An approximate (isotropic) treatment of cell e.s.d.'s is used for estimating e.s.d.'s involving l.s. planes.
Refinement. Refinement of *F*^2^ against ALL reflections. The weighted *R*-factor *wR* and goodness of fit *S* are based on *F*^2^, conventional *R*-factors *R* are based on *F*, with *F* set to zero for negative *F*^2^. The threshold expression of *F*^2^ > σ(*F*^2^) is used only for calculating *R*-factors(gt) *etc*. and is not relevant to the choice of reflections for refinement. *R*-factors based on *F*^2^ are statistically about twice as large as those based on *F*, and *R*- factors based on ALL data will be even larger.

### Fractional atomic coordinates and isotropic or equivalent isotropic displacement parameters (Å^2^)

**Table:**

	*x*	*y*	*z*	*U*_iso_*/*U*_eq_	Occ. (<1)
N1	0.2185 (2)	−0.0996 (7)	0.16024 (12)	0.0654 (8)	
O1	0.24369 (16)	−0.2609 (7)	0.10176 (10)	0.0807 (7)	
O2	0.09872 (13)	0.1007 (6)	0.23812 (10)	0.0734 (7)	
H2	0.1157	0.0056	0.2054	0.110*	
C1	0.1626 (2)	−0.4239 (10)	0.07147 (17)	0.0768 (10)	
H1A	0.1376	−0.5343	0.1086	0.092*	
H1B	0.1829	−0.5633	0.0379	0.092*	
C2	0.0834 (2)	−0.2382 (10)	0.03427 (15)	0.0700 (9)	
H2A	0.1085	−0.1218	−0.0018	0.084*	
H2B	0.0606	−0.1049	0.0681	0.084*	
C3	0.0000	−0.4226 (13)	0.0000	0.0661 (13)	
H3A	−0.0223	−0.5478	0.0357	0.079*	0.50
H3B	0.0223	−0.5478	−0.0357	0.079*	0.50
C4	0.3854 (2)	0.0583 (12)	0.16832 (16)	0.0890 (13)	
H4A	0.3863	−0.0668	0.1279	0.134*	
H4B	0.4321	−0.0087	0.2061	0.134*	
H4C	0.4004	0.2552	0.1559	0.134*	
C5	0.2876 (2)	0.0510 (9)	0.19192 (15)	0.0621 (9)	
C6	0.1752 (2)	0.2479 (9)	0.27207 (14)	0.0543 (8)	
C7	0.2658 (2)	0.2281 (8)	0.25198 (13)	0.0527 (8)	
C8	0.3400 (2)	0.3899 (9)	0.29307 (16)	0.0673 (10)	
H8	0.4022	0.3771	0.2816	0.081*	
C9	0.3230 (2)	0.5610 (10)	0.34806 (16)	0.0710 (10)	
H9	0.3731	0.6660	0.3728	0.085*	
C10	0.2308 (2)	0.5826 (8)	0.36834 (14)	0.0575 (8)	
C11	0.1552 (2)	0.4251 (8)	0.32992 (14)	0.0533 (7)	
C12	0.0631 (2)	0.4448 (10)	0.35059 (15)	0.0704 (10)	
H12	0.0128	0.3420	0.3255	0.084*	
C13	0.0467 (3)	0.6102 (10)	0.40623 (17)	0.0794 (11)	
H13	−0.0145	0.6170	0.4198	0.095*	
C14	0.1205 (3)	0.7717 (11)	0.44378 (16)	0.0804 (11)	
H14	0.1083	0.8883	0.4817	0.097*	
C15	0.2101 (3)	0.7583 (10)	0.42496 (17)	0.0722 (10)	
H15	0.2589	0.8676	0.4501	0.087*	

### Atomic displacement parameters (Å^2^)

**Table:**

	*U*^11^	*U*^22^	*U*^33^	*U*^12^	*U*^13^	*U*^23^
N1	0.0693 (18)	0.071 (2)	0.0551 (13)	0.0114 (18)	0.0037 (13)	0.0008 (16)
O1	0.0745 (15)	0.0976 (19)	0.0678 (13)	0.0151 (17)	0.0001 (12)	−0.0131 (16)
O2	0.0503 (12)	0.0963 (19)	0.0721 (13)	−0.0068 (14)	0.0014 (10)	−0.0109 (14)
C1	0.090 (2)	0.067 (2)	0.0693 (19)	0.015 (3)	−0.0063 (19)	−0.006 (2)
C2	0.080 (2)	0.066 (2)	0.0609 (17)	0.006 (2)	−0.0040 (16)	−0.003 (2)
C3	0.081 (3)	0.061 (3)	0.057 (2)	0.000	0.012 (2)	0.000
C4	0.066 (2)	0.132 (4)	0.071 (2)	0.001 (3)	0.0156 (17)	−0.012 (3)
C5	0.0552 (19)	0.077 (2)	0.0519 (17)	0.010 (2)	−0.0001 (15)	0.0130 (19)
C6	0.0465 (17)	0.065 (2)	0.0489 (15)	0.0020 (19)	−0.0041 (13)	0.0072 (18)
C7	0.0489 (18)	0.062 (2)	0.0458 (15)	0.0028 (18)	−0.0001 (13)	0.0135 (17)
C8	0.0472 (18)	0.088 (3)	0.0649 (19)	−0.006 (2)	0.0019 (15)	0.007 (2)
C9	0.054 (2)	0.090 (3)	0.0660 (19)	−0.014 (2)	−0.0010 (16)	0.000 (2)
C10	0.0544 (18)	0.065 (2)	0.0518 (16)	0.0001 (19)	0.0018 (15)	0.0119 (19)
C11	0.0501 (17)	0.060 (2)	0.0490 (15)	0.0016 (17)	0.0035 (13)	0.0130 (18)
C12	0.055 (2)	0.095 (3)	0.0608 (18)	−0.001 (2)	0.0049 (15)	0.001 (2)
C13	0.067 (2)	0.100 (3)	0.073 (2)	0.008 (2)	0.0121 (19)	0.002 (2)
C14	0.089 (3)	0.090 (3)	0.0620 (19)	0.008 (3)	0.011 (2)	−0.003 (2)
C15	0.074 (2)	0.077 (3)	0.0620 (18)	−0.009 (2)	−0.0031 (17)	0.004 (2)

### Geometric parameters (Å, °)

**Table:**

N1—C5	1.278 (4)	C5—C7	1.465 (5)
N1—O1	1.417 (3)	C6—C7	1.380 (4)
O1—C1	1.422 (4)	C6—C11	1.424 (4)
O2—C6	1.360 (3)	C7—C8	1.428 (4)
O2—H2	0.8200	C8—C9	1.352 (4)
C1—C2	1.503 (4)	C8—H8	0.9300
C1—H1A	0.9700	C9—C10	1.402 (4)
C1—H1B	0.9700	C9—H9	0.9300
C2—C3	1.522 (5)	C10—C15	1.404 (5)
C2—H2A	0.9700	C10—C11	1.410 (4)
C2—H2B	0.9700	C11—C12	1.404 (4)
C3—C2^i^	1.522 (5)	C12—C13	1.345 (5)
C3—H3A	0.9700	C12—H12	0.9300
C3—H3B	0.9700	C13—C14	1.395 (5)
C4—C5	1.501 (4)	C13—H13	0.9300
C4—H4A	0.9600	C14—C15	1.355 (4)
C4—H4B	0.9600	C14—H14	0.9300
C4—H4C	0.9600	C15—H15	0.9300
			
C5—N1—O1	113.5 (3)	O2—C6—C7	122.6 (3)
N1—O1—C1	108.8 (2)	O2—C6—C11	115.3 (2)
C6—O2—H2	109.5	C7—C6—C11	122.2 (3)
O1—C1—C2	113.8 (3)	C6—C7—C8	116.8 (3)
O1—C1—H1A	108.8	C6—C7—C5	123.1 (3)
C2—C1—H1A	108.8	C8—C7—C5	120.1 (3)
O1—C1—H1B	108.8	C9—C8—C7	122.3 (3)
C2—C1—H1B	108.8	C9—C8—H8	118.9
H1A—C1—H1B	107.7	C7—C8—H8	118.9
C1—C2—C3	112.0 (3)	C8—C9—C10	121.0 (3)
C1—C2—H2A	109.2	C8—C9—H9	119.5
C3—C2—H2A	109.2	C10—C9—H9	119.5
C1—C2—H2B	109.2	C9—C10—C15	122.8 (3)
C3—C2—H2B	109.2	C9—C10—C11	119.0 (3)
H2A—C2—H2B	107.9	C15—C10—C11	118.2 (3)
C2—C3—C2^i^	112.9 (5)	C12—C11—C10	119.0 (3)
C2—C3—H3A	109.0	C12—C11—C6	122.3 (3)
C2^i^—C3—H3A	109.0	C10—C11—C6	118.8 (3)
C2—C3—H3B	109.0	C13—C12—C11	121.0 (3)
C2^i^—C3—H3B	109.0	C13—C12—H12	119.5
H3A—C3—H3B	107.8	C11—C12—H12	119.5
C5—C4—H4A	109.5	C12—C13—C14	120.6 (3)
C5—C4—H4B	109.5	C12—C13—H13	119.7
H4A—C4—H4B	109.5	C14—C13—H13	119.7
C5—C4—H4C	109.5	C15—C14—C13	119.8 (4)
H4A—C4—H4C	109.5	C15—C14—H14	120.1
H4B—C4—H4C	109.5	C13—C14—H14	120.1
N1—C5—C7	116.3 (3)	C14—C15—C10	121.4 (4)
N1—C5—C4	122.8 (3)	C14—C15—H15	119.3
C7—C5—C4	120.9 (3)	C10—C15—H15	119.3
			
C5—N1—O1—C1	178.9 (3)	C8—C9—C10—C15	−179.8 (4)
N1—O1—C1—C2	69.9 (4)	C8—C9—C10—C11	−0.7 (5)
O1—C1—C2—C3	177.5 (2)	C9—C10—C11—C12	179.5 (3)
C1—C2—C3—C2^i^	176.6 (3)	C15—C10—C11—C12	−1.4 (5)
O1—N1—C5—C7	178.7 (3)	C9—C10—C11—C6	0.4 (4)
O1—N1—C5—C4	0.9 (5)	C15—C10—C11—C6	179.5 (3)
O2—C6—C7—C8	−178.8 (3)	O2—C6—C11—C12	0.3 (5)
C11—C6—C7—C8	1.3 (5)	C7—C6—C11—C12	−179.8 (3)
O2—C6—C7—C5	0.9 (5)	O2—C6—C11—C10	179.4 (3)
C11—C6—C7—C5	−179.0 (3)	C7—C6—C11—C10	−0.7 (5)
N1—C5—C7—C6	−3.4 (5)	C10—C11—C12—C13	−0.2 (5)
C4—C5—C7—C6	174.5 (4)	C6—C11—C12—C13	178.9 (3)
N1—C5—C7—C8	176.3 (3)	C11—C12—C13—C14	1.5 (6)
C4—C5—C7—C8	−5.8 (5)	C12—C13—C14—C15	−1.2 (6)
C6—C7—C8—C9	−1.6 (5)	C13—C14—C15—C10	−0.4 (6)
C5—C7—C8—C9	178.6 (3)	C9—C10—C15—C14	−179.2 (4)
C7—C8—C9—C10	1.4 (5)	C11—C10—C15—C14	1.7 (6)

Symmetry codes: (i) −*x*, *y*, −*z*.

### Hydrogen-bond geometry (Å, °)

**Table:**

*D*—H···*A*	*D*—H	H···*A*	*D*···*A*	*D*—H···*A*
O2—H2···N1	0.82	1.83	2.552 (3)	145
